# A simple and affordable method for estimating the fluid volume a mosquito sucks using food dyes

**DOI:** 10.1186/s41182-021-00302-6

**Published:** 2021-02-03

**Authors:** Chisako Sakuma, Hirotaka Kanuka

**Affiliations:** 1 Department of Tropical Medicine, The Jikei University School of Medicine, Tokyo, Japan; 2 Center for Medical Entomology, The Jikei University School of Medicine, Tokyo, Japan

**Keywords:** Food dye, ATP, Membrane feeding, *Aedes aegypti*, Mosquitoes, Capsaicin

## Abstract

**Background:**

Blood-sucking by mosquitoes is an inevitable behavior when pathogens are transmitted among humans. Adenine nucleotides such as ATP are known as phagostimulants for mosquitoes and are widely used to induce and enhance the blood-sucking activity in an artificial manner. Although using ATP solution is convenient to introduce a variety of substances (for example chemicals and pathogens) into the mosquito body via sucking, establishing an easy and cost-effective method to quantify the amount of solution ingested has yet to be reported.

**Results:**

A set of commercial food dyes (green, blue, yellow, and red) was employed in this study. Each dye was added to ATP solution used to colorize the abdomen of *Ae. aegypti* female mosquitoes after ingestion. The intake of food dyes did not show any toxicity to the mosquitoes, affecting neither ATP-sucking behavior nor survival of the mosquitoes. We observed that quantifying the color intensity of green dye in the mosquito abdomen by spectral analysis, as well as distinguishing the size of the colored abdomen using the naked eye, allowed the estimation of the amount of ingested solution. Using this method, capsaicin, a pungent component of chili peppers, was identified as an aversive tastant that can discourage mosquitoes from sucking the ATP solution.

**Conclusions:**

Employing commercially available, non-toxic food dyes converted ATP-driven membrane feeding into an easy-to-use method to estimate the amount of solution ingested by mosquitoes. This method can be further applied for a variety of experiments such as introducing a certain quantity of chemical compounds or microbes into the mosquito body.

**Supplementary Information:**

The online version contains supplementary material available at 10.1186/s41182-021-00302-6.

## Background

*Aedes aegypti* is the primary vector species spreading a number of arboviral diseases, such as Zika and dengue fever [1]. The blood-sucking behavior contributes to transmission cycle of these viruses between mosquitoes and vertebrate hosts [2, 3]. The viral particles circulating in the blood of infected hosts are ingested into the midgut of mosquitoes as a blood meal. The virus invades into epithelial cells of the midgut and spreads systemically through hemocoel to other tissues including salivary glands. Saliva excreted from the salivary gland transfers virus particles into the new host during a bite [2, 3]. How much blood meal is ingested affects the infection rate of pathogens in each mosquito [4]. Furthermore, mosquito fecundity is influenced by the quality and quantity of a blood meal, mainly providing essential nutrients such as amino acids for vitellogenesis and oogenesis [5]. Thus, it is crucial to reveal the whole process and underlying mechanisms of blood-sucking behavior toward understanding hematophagy in arthropods and developing effective vector control strategies.

It is widely known that adenine nucleotides in the blood stimulate the blood-sucking behavior of mosquitoes [6]. Adenosine triphosphate (ATP) is the most potent stimulant among adenine nucleotides especially in *Ae. aegypti* [7]. From a series of studies to maximize the phagostimulus effect of adenine nucleotides, the optimum concentrations of NaCl and NaHCO_3_ were identified to elevate the gorging responses of *Ae. aegypti* [8, 9]. In accordance with the evaluation of artificial phagostimulants, membrane-feeding methods have also been developed; a variety type of membranes, bloods of animal species, and multiple designs of feeders were examined [10–15]. One of the outstanding advantages of membrane feeding instead of using live animals is to modify the constitution of solution to be ingested by mosquitoes. For example, membrane feeding allows researchers to introduce a specific amount of pathogens such as viruses into the mosquito body [16]. It also enables investigation of the effect of any particular molecules on the physiology of mosquitoes such as longevity, metabolism, and reproduction by adding these molecules into the membrane feeder [8].

Although membrane feeding is an easy-to-use and multipurpose method for mosquito studies, it still remains a critical problem that it is difficult to quantify the amount of blood/ATP solution in a mosquito’s body post membrane feeding. One of the conventional methods to estimate how much solution a mosquito takes in is to weigh the mosquito before and after feeding [17]. The weight of a starved mosquito is approximately 2–3 mg, and it reaches 6–7 mg after being fully engorged. However, the gravimetric method usually requires a fine and expensive electronic microbalance that can precisely measure the weight of one mosquito in the order of micrograms.

To make quantification convenient and accurate, a variety of labeling substances have been introduced. A method using radioisotope (^144^Ce)-labeled blood and gamma counting proved useful and reliable based on the fact that the radioisotope is not excreted in the urine [18], whereas radioisotope methods are less accessible in the general laboratory. A hemoglobinometry method using cyanide has been applied for blood measuring in mosquitoes, in which complete conversion of all hemoglobin from lysed erythrocytes to a stable complex (hemiglobincyanide) occurs [17]. Adding fluorescent materials such as fluorescein into blood meals has also been reported [19, 20]. However, all of these methods require the mosquitoes to be killed for the quantification process, making it difficult to continue observing mosquitoes for further experiments.

In this study, we adopted a set of food dyes manufactured by a food company for membrane feeding, and we observed that these dyes effectively colorized the abdomens of mosquitoes when ingested. The color intensity presented in mosquitoes was quantitatively measured using a spectrometer and also distinguished using the naked eye. The ingested dyes did not affect either taste perception or viability of the mosquitoes, allowing researchers to perform further experiments with these mosquitoes.

## Methods

### Mosquito rearing

The *Ae. aegypti* strain used in this study, which originated from the Liverpool strain, was a gift from Dr. Ryuichiro Maeda (Obihiro University of Agriculture and Veterinary Medicine). Eggs obtained using a standard procedure were hatched in reverse osmosis (RO) water. The first instar larvae were transferred to a plastic container with RO water and daily fed with fish food (Hikari, #4971618-013378, Kyorin Co., Ltd., Japan). The larvae were kept in an insectary room set at 27 °C, and water in the container was refreshed every 2–3 days. The pupae were harvested in a plastic cup and placed within a cage (bottom 27 cm × 27 cm, top 25 cm × 25 cm, height 27 cm), in which a 50-ml glass flask containing 10% sucrose solution with a filter paper inserted (#1001-125, Whatman, USA) was placed. The cage rearing the emerged adults was kept in an incubator (MIR254-PJ, Panasonic Co., Japan) set at 27 °C with humidity over 90% in a standard 12 h:12 h light-dark cycle. The sucrose solution was changed every 3–4 days.

### Membrane feeding

Four commercial food dyes (green: #4901325-000484, blue: #49861457, yellow: #4901325-001146, red: #4901325-001245, Kyoritsu Food Co. Inc., Japan) were dissolved in ultrapure water (500 μg/μl). One hundred millimolars ATP solution was purchased (#BSA04-001, Cytoskeleton Inc., USA). Capsaicin (#034-11351, Wako Co., Japan) was purchased and dissolved in 95% EtOH to prepare 1 mM solution as stock. About thirty female adult mosquitoes (at 5–7 days after eclosion) were collected using an aspirator and transferred into a paper cup (top diameter 97.2 mm × height 100 mm × bottom diameter 74.3 mm) (#31-476-001, Tokan Kogyo Co., Ltd., Japan) covered with a drawing net. The paper cup was kept in an incubator set at 27 °C for 18 h without sucrose solution. A working solution for membrane feeding (0.1 or 1 mM ATP, 4 or 20 μg/μl of food dye, 150 mM NaCl, 10 mM NaHCO_3_ in ultrapure water) was prepared immediately before the experiment and applied to mosquito feeders (#CG-1835-70, Chemglass Life Sciences, USA), the bottom of which was covered with a sheet of parafilm (#BR701605, Merck, Germany). Each feeder was then placed on the paper cup containing mosquitoes and circulated with 42 °C water from a hot water bath coupled with a water pump (FKP-1, AS ONE Corp., Japan) for 10 min (see Fig. S1). After feeding, the mosquitoes in each paper cup were sacrificed in the freezer or anesthetized using carbon dioxide for further experiments. Compositions of the solutions used for membrane feeding in this study are listed in Table S1.

### Longevity assay

To examine mosquito life-span, mosquitoes were anesthetized immediately after membrane feeding using carbon dioxide, and only fully engorged mosquitoes were collected. Twenty to thirty engorged females were transferred to a new paper cup. Sucrose solution (10%) was supplied with a cotton pad placed on the top of the cup and changed every 3–4 days. Each paper cup was kept in an incubator set at 27 °C with humidity over 90% in cycles of 12 h light and 12 h darkness. The number of living mosquitoes was counted every day.

### Quantification of dye by spectral analysis

To determine the optimal wavelength for measuring the commercial food dye, we selected the green dye and scanned its absorbance spectra using 100 μg/μl solution using a multilabel plate reader (EnSpire 2300, Perkin Elmer Inc., USA). The scanned data indicated that a wavelength of 638 nm is supposed to be optimal for measuring the green dye.

To determine how much dye is ingested by a mosquito, each mosquito colorized with green dye was transferred to a 1.5-ml tube containing 230 μl of PBS, and lightly homogenized with three strokes of a loose-fitting pestle (#96.07339.9.03, Treff Lab, Switzerland). The homogenized mixture was centrifuged at 20379 *g* for 10 min at room temperature, and then 200 μl of supernatant was transferred to a 96-well plate. The absorbance of each supernatant was measured at 638 nm by the plate reader.

### Imaging of colorized mosquitoes

To perform image analysis of dye-fed mosquitoes, each mosquito harboring green dye in its abdomen was sacrificed by freezing for 10 min and images recorded using a binocular microscope equipped with a digital camera (α5100, Sony Co., Japan). Each mosquito was graded into one of four classes by gross classification of both size and color of its abdomen (class 1A, fully engorged abdomen; class 1B, colored, partially swollen abdomen; class 2, colored abdomen with intact shape; class 3, abdomen with intact shape and no color observed). Thickness of the mosquito abdomen was measured along the dorso-ventral axis using the Adobe Photoshop CS3 Extended image processing software (version 10.0.1, Adobe Inc., USA). Briefly, each image was rotated to allocate the anterior–posterior axis of a mosquito in a horizontal position. Subsequently, the maximum thickness of the abdomen was measured using the Ruler tool of the software.

### Statistical analysis

Dunnett’s multiple comparison test, Kaplan–Meier log-rank (Mantel–Cox) test, simple linear regression analysis, and chi-square test were performed in this study using Prism 8 (GraphPad Software, USA). Probability values less than 0.05 were considered statistically significant. Raw data of all experiments is available in Table S2.

## Results

### Effective colorization of mosquito abdomen by food dyes in ATP-based membrane feeding

**Fig. 1:**
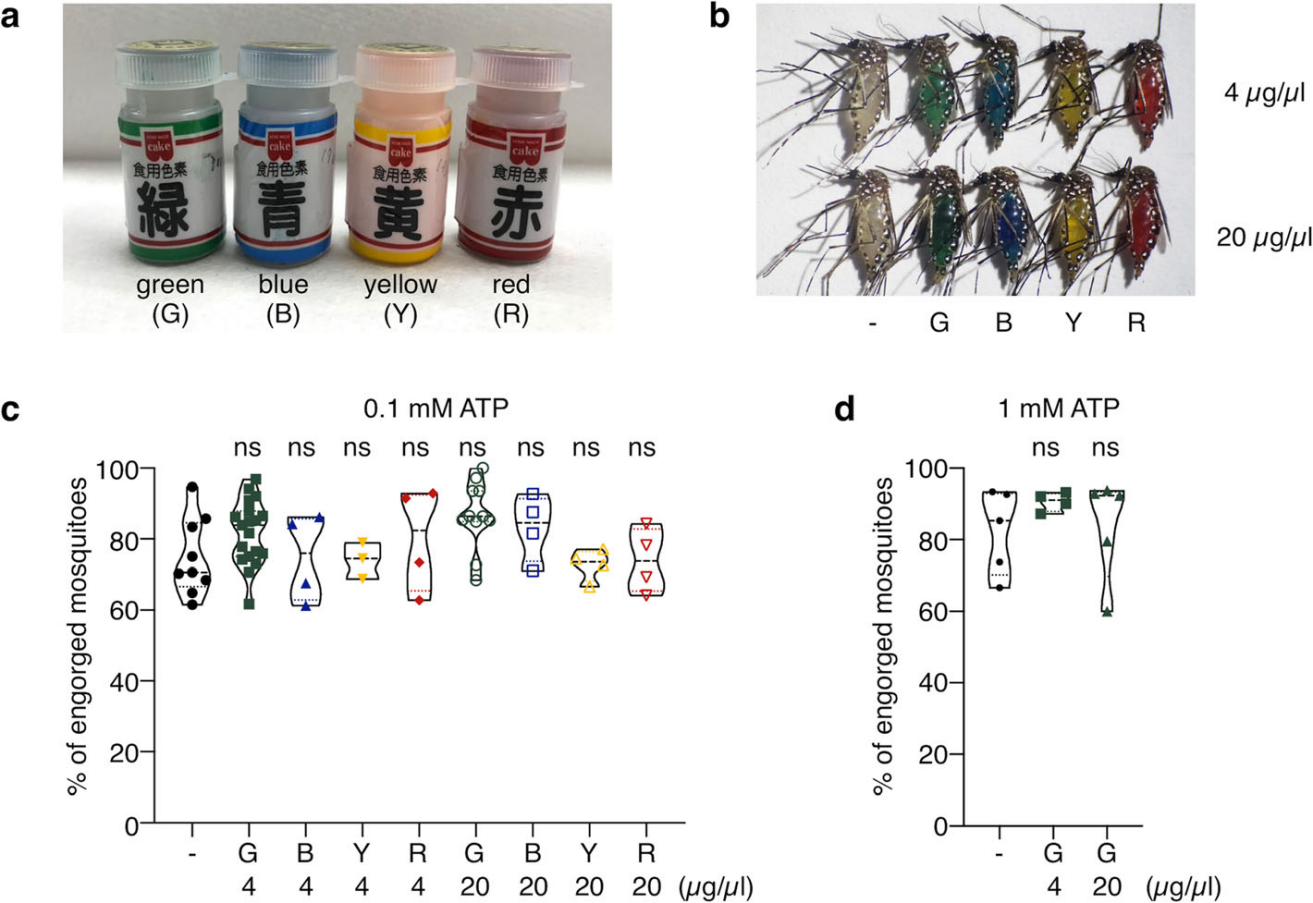
Food dyes colorize mosquito abdomen using ATP-based membrane feeding. **a** Food dyes used in this study. **b** A representative example of *Ae. aegypti* female mosquitoes that sucked 0.1 mM ATP solution containing 4 μg/μl (upper row) or 20 μg/μl (lower row) of each food dye. -, G, B, Y, and R indicate no food dye (control), green, blue, yellow, and red, respectively. **c**, **d** *Ae. aegypti* female mosquitoes engorged with 0.1 mM (**c**) or 1 mM ATP (**d**) solution containing the indicated concentration of each food dye. All experiments were performed more than three times. Dunnett’s multiple comparison test; ns, not significant

To improve the membrane feeding method capable of quantitatively measuring the amount of solution ingested by mosquitoes, we considered optical dye as a tool to label the solution. A set of commercially available food dyes (green, blue, yellow, and red) was employed (Fig. 1a). *Ae. aegypti* female mosquitoes were subjected to membrane feeding with 0.1 mM ATP solutions containing 4 or 20 μg/μl of each food dye (Fig. S1). As a result, we observed that the abdomens of the mosquitoes that ingested each colorized ATP solution were visibly colorized (Fig. 1b). No significant difference was observed in the proportion of the engorged mosquitoes among the food dyes tested (Fig. 1c, *p* = 0.0635 for no dye vs. 20 μg/μl green dye and *p* > 0.4 for other comparisons), indicating that these dyes did not affect the perception of taste (ATP), attractiveness to the artificial target (warm water), or sucking speed. When a higher concentration of ATP (1 mM) was used for membrane feeding, the green dye (4 and 20 μg/μl) did not prevent the mosquitoes from being engorged (Fig. 1d, *p* = 0.4738 for 0 μg/μl vs. 4 μg/μl, *p* = 0.9738 for 0 μg/μl vs. 20 μg/μl).

### Influence of ingesting food dyes on survival and longevity of mosquitoes

**Fig. 2:**
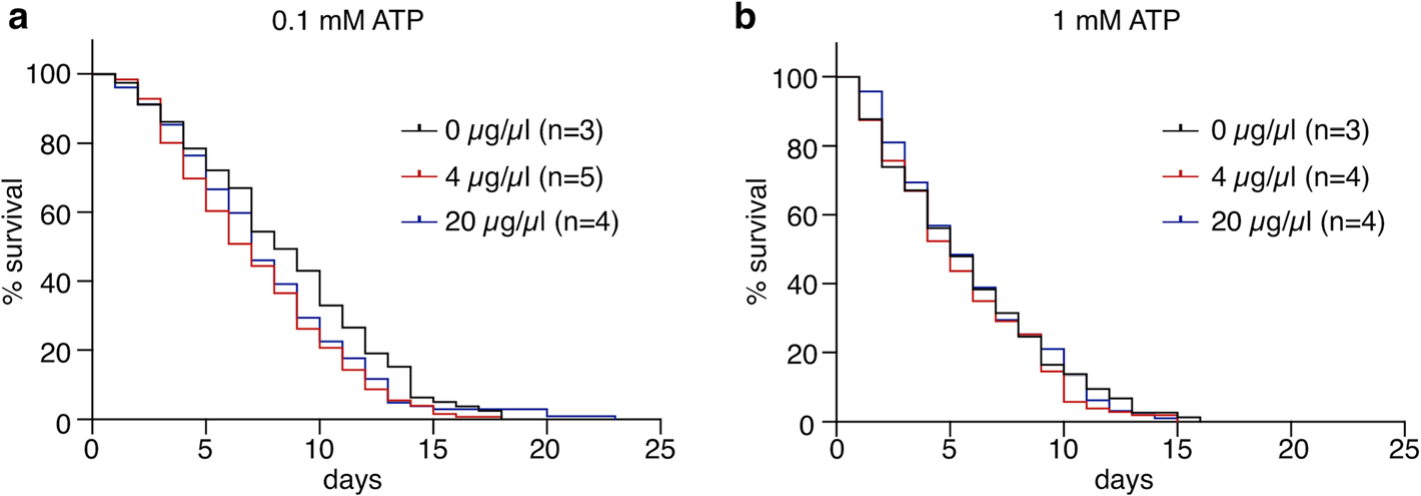
Influence of ingesting food dyes on survival and longevity of mosquitoes. **a**, **b** Survival rate of *Ae. aegypti* female mosquitoes that sucked 0.1 mM (**a**) or 1 mM (**b**) ATP solution containing either 4 μg/μl or 20 μg/μl green dye. The survival rate of 20–30 engorged mosquitoes was followed each day after membrane feeding. All experiments were performed more than three times. No significant difference was found in the Kaplan–Meier log-rank (Mantel-Cox) test (0.1 mM ATP, *p* = 0.0576; 1 mM ATP, *p* = 0.5808)

Given that experiments such as observing pathogen dynamics or blood-sucking behavior usually take a certain period of time, the food dyes were expected not to impair the physiological conditions of mosquitoes. To examine whether the life span of mosquitoes may be affected by ingesting the food dyes, we counted the number of living *Ae. aegypti* adult females each day after feeding of ATP solution with 4 or 20 μg/μl green dye. No significant difference in longevity was observed between the dye-ingested and control mosquitoes (Fig. 2a, b, 0.1 mM ATP, *p* = 0.0576; 1 mM ATP, *p* = 0.5808). These results provided us the further opportunity to develop an improved, quantitative method for membrane feeding using the food dyes as follows.

### Colorimetric quantification of ATP solution ingested by mosquito

**Fig. 3:**
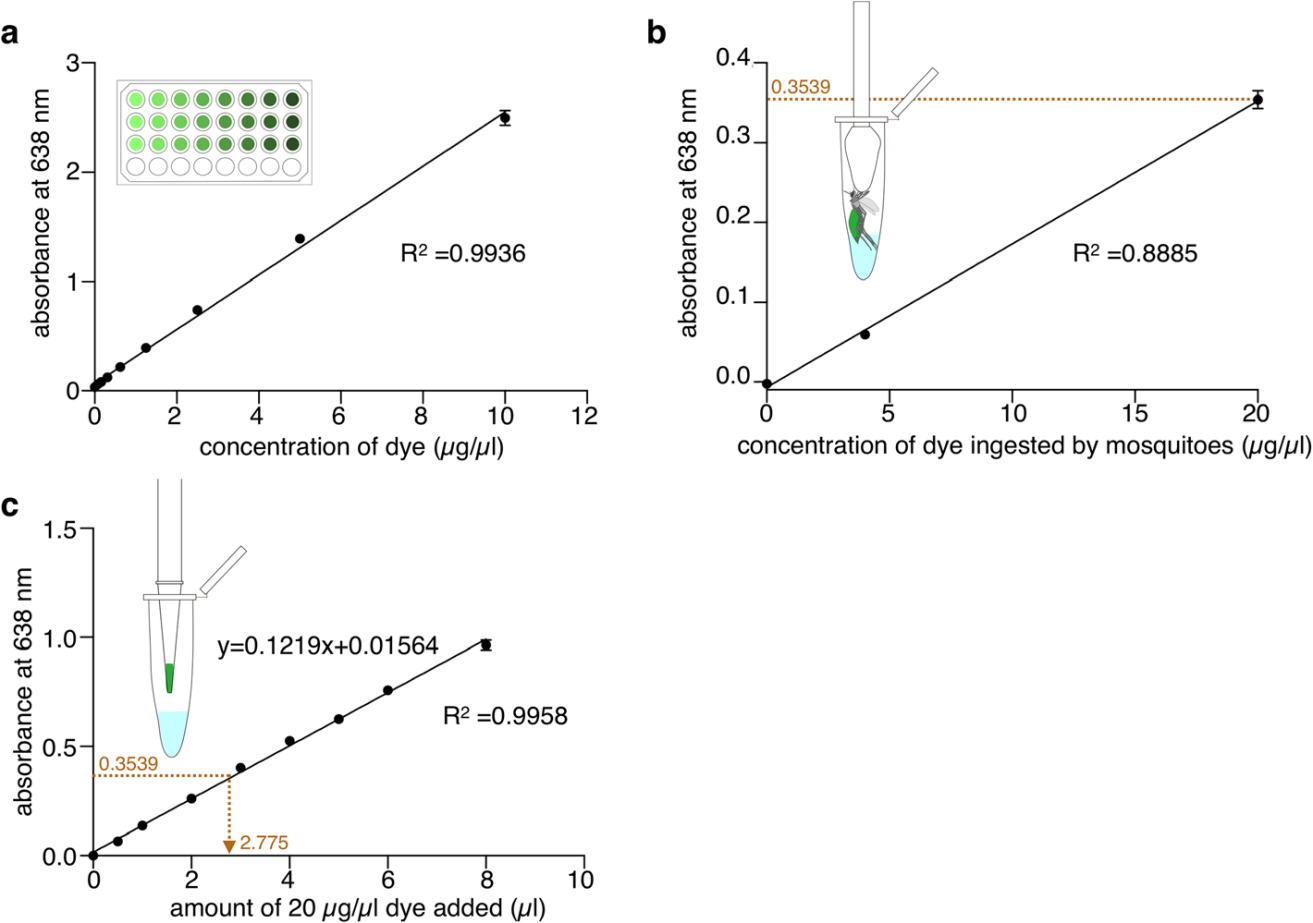
Colorimetric quantification of ATP solution ingested by mosquitoes. **a** Absorbance at 638 nm of two-fold serial dilutions of green dye which was dissolved in ultrapure water. Two hundred microliter of each solution containing the indicated concentration of green dye was applied to a well of 96-well plate. Simple linear regression analysis was performed (*R*^2^ = 0.9936). **b** Absorbance at 638 nm of homogenized solution prepared from each *Ae. aegypti* female mosquito that sucked 0.1 mM ATP solution containing either 0 μg/μl, 4 μg/μl, or 20 μg/μl of green dye. All experiments were performed more than three times. The numbers of mosquitoes used at each concentration of dye were 89, 83, and 74, respectively. All error bars show standard error of mean (SEM), and two bars in this figure were not visible due to the small values of SEM. Simple linear regression analysis was performed (*R*^2^ = 0.8885). The average absorbance of mosquitoes fed with ATP solution (20 μg/μl green dye) was 0.3539. **c** Absorbance at 638 nm of PBS containing green dye. Each aliquot (0.5–8 μl) of 20 μg/μl green dye was added to a 1.5-ml tube containing 230 μl of PBS, and then 200 μl of each solution was transferred to a 96-well plate. All error bars show SEM. Simple linear regression derived a formula to estimate how much volume of ATP solution a mosquito sucks

To obtain information about how much fluid a mosquito intakes into the abdomen, first, we determined the optimal wavelength for measuring absorbance of the green dye used in this study. The absorption spectrum of the green dye showed one of the broad peaks of absorption corresponding to a maximum around 638 nm (see “Methods”), followed by measuring the absorbance of serial dilution of the dye at 638 nm (Fig. 3a). Supernatants prepared from homogenized mosquitoes that were fed with ATP solution containing 4 or 20 μg/μl green dye were then analyzed, resulting that the absorbance at 638 nm was also proportional to the concentration of green dye even after the dye was ingested by mosquitoes (Fig. 3b). Simple linear regression analysis with the result of measured absorbance of diluted green dye (20 μg/μl) derived a formula to estimate the volume of the solution ingested by a mosquito (Fig. 3c). In the case of the mosquitoes fed on the ATP solution containing 20 μg/μl green dye (as shown in Fig. 3b), the estimated volume was calculated as 2.775 μl, in agreement with previous reports [17, 19, 20].

### Estimation of the volume of ATP solution in mosquito by naked eye

**Fig. 4:**
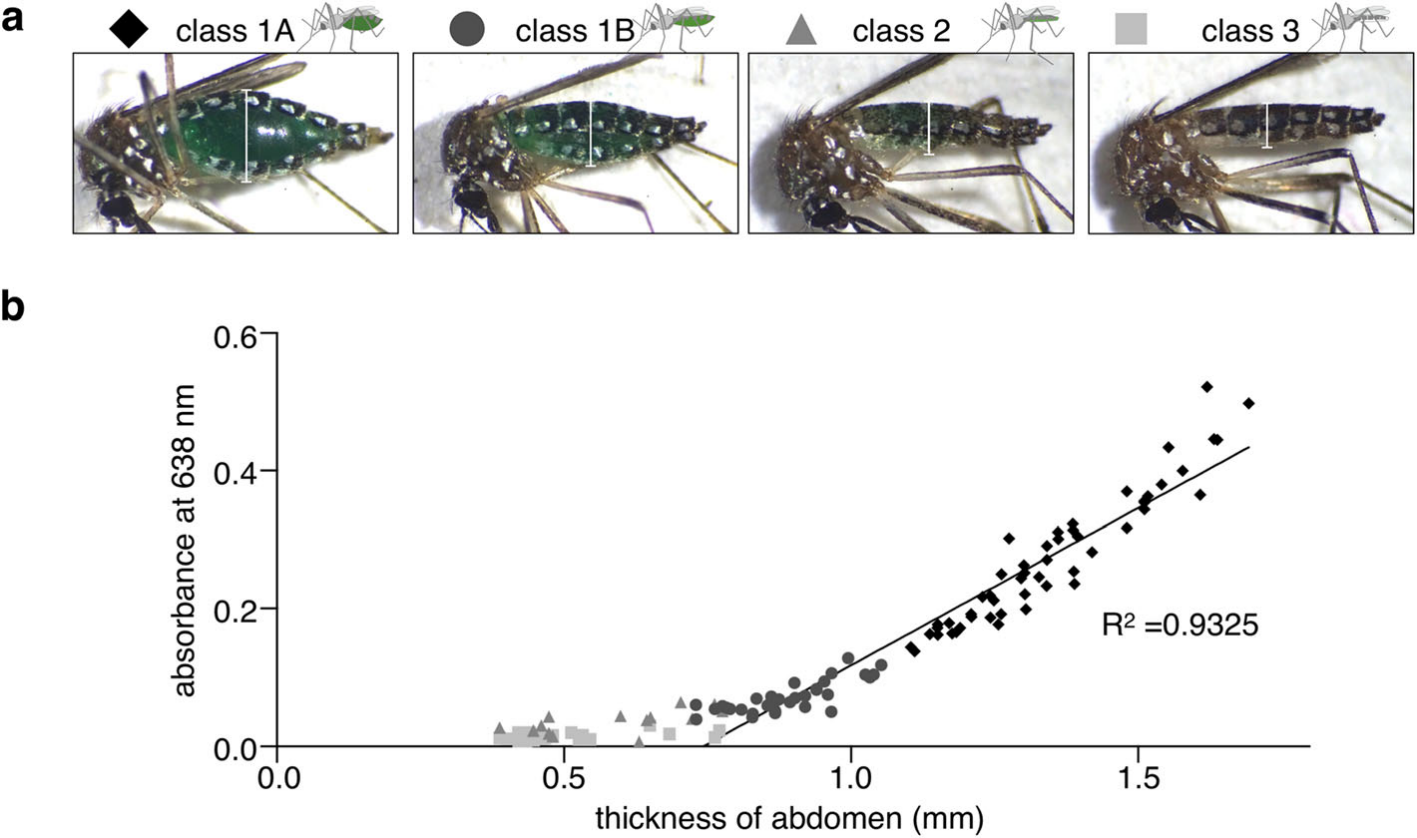
Estimation of volume of ATP solution ingested by mosquitoes using the naked eye. **a** Classification of mosquitoes that sucked green-colored 0.1 mM ATP solution. Class 1A (diamond), fully engorged abdomen; class 1B (circle), partially swollen abdomen; class 2 (triangle), colored abdomen with intact shape; and class 3 (square), abdomen with intact shape and no color observed. White vertical lines on each image indicate the thicknesses of abdomens. **b** A scatter plot indicates a linear relationship between the two variables, absorbance at 638 nm of the mosquito extract and the thicknesses of mosquito abdomens. Simple linear regression analysis was performed using data of class 1A and 1B mosquitoes (*R*^2^ = 0.9325)

The colored abdomen of a mosquito is easily visible to the unaided eye (Fig. 1b). To investigate whether the food dye helped us estimate the approximate amount of ingested fluid by gross morphology, a number of the mosquitoes that sucked varying amounts of ATP solution containing 20 μg/μl green dye were prepared by altering the duration of sucking time in membrane feeding. These mosquitoes were then divided into four classes according to the color and shape of their abdomens (see “Methods”) (Fig. 4a). Each mosquito was homogenized to conduct spectral analysis of the ingested green dye, following the thickness of each mosquito abdomen being determined. As a result, the absorbance at 638 nm and the thickness of the abdomen were proportional to each other (*R*^2^ = 0.9325), and the gross classification of ingested mosquitoes correlated well with these two parameters (Fig. 4b). These results suggested that naked eye-based estimating how much a mosquito sucked the green-colored solution may be sufficient for practical use.

### Identification of the aversive action of capsaicin on feeding behavior of mosquitoes

**Fig. 5:**
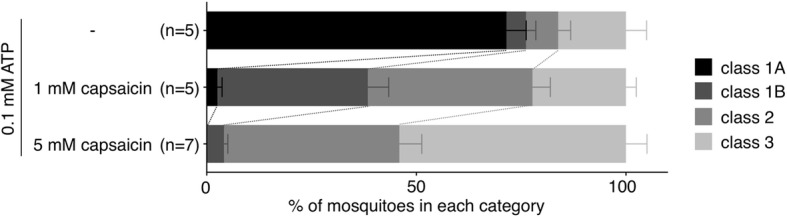
Aversive action of capsaicin on feeding behavior of mosquitoes. Thirty to forty *Ae. aegypti* female mosquitoes that sucked 0.1 mM ATP solution containing 1 mM or 5 mM capsaicin were classified into four classes as shown in Fig. 4a: class 1A (black), class 1B (gray), class 2 (light gray), class 3 (gray-white). All experiments were performed more than five times. Chi-square test was performed

We assumed that the method to estimate the fluid volume sucked in mosquitoes using the naked eye with the support of the colorizing solution may be applicable for identifying molecules that potentially affect blood-sucking behavior. It has been widely conceived that ecdysozoans including flies and nematodes, unlike mammals, do not perceive capsaicin, a chemical irritant [21]. On the other hand, *Drosophila melanogaster* showed a preference for capsaicin in a dose-dependent manner when exposed to a choice between capsaicin-containing sucrose and sucrose alone [22]. To examine whether mosquitoes prefer or avoid sucking capsaicin, *Aedes* female mosquitoes fed with green-colorized ATP solution containing 1 mM or 5 mM capsaicin were grossly classified into four classes by color and shape of the abdomens (Fig. 5). The number of fully engorged mosquitoes (class 1A) was extremely decreased even when treated with low dose of capsaicin, and accordingly, the rate of mosquitoes with green abdomens and no shape change (class 2) was significantly increased (chi-square test, *p* < 0.001). These results suggested that capsaicin is aversive to mosquitoes, exerting a negative impact on feeding behavior induced by the phagostimulant molecule ATP.

## Discussion

In this study, we adopted food dyes with ATP-stimulated membrane feeding, which effectively colorized the abdomens of mosquitoes. Each dye did not affect the physiology of mosquitoes and made it possible to estimate the amount of ingested ATP solution even using the naked eye in addition to spectral analysis.

There are multiple alternatives for labeling the solution that can be loaded into a membrane feeder, such as radioisotopes and fluorescent substances. These materials, however, require expensive, specialized equipment for measuring radioactivity and fluorescent intensity. On the other hand, using dyes had a number of benefits; the dyes can be excited by visible light and are relatively affordable and applicable for many purposes. Among a wide variety of dyes, we employed edible dyes that are normally used as food coloring as agents to label the ATP solution because food dyes are comparatively non-toxic and have already satisfied the criteria of food safety standards. For example, the green dye we used in this study consisted of Tartrazine (8.6%) and Brilliant Blue FCF (3.4%), which are the approved food additives. Other commercially available food dyes were also used in artificial food, which can become a substitute for animal blood to rear mosquitoes, showing that these food dyes do not exhibit any negative effects on the mosquito life cycle for many generations [23, 24]. The fact that most food dyes are easily accessible may expand the availability of membrane feeding of mosquitoes to a range of other experiments.

Applying dyes to conventional methods for membrane feeding may offer several important advantages to researchers. First, using the dyes provides a simplified way to distinguish mosquitoes that ingest the ATP solution from non-sucked ones even when mosquitoes intake a small amount of the solution. It is also possible to discriminate mosquitoes with slightly swollen abdomens that do not carry any amount of the solution. As shown in Fig. 4 a and b (grey squares), the thicknesses of abdomens varied between individuals at 18 h after starvation. Second, it can be achieved with the combination of membrane feeding with dye to track each mosquito for a certain period of time (even until end of life-span) after estimating the volume of ingested solution using the naked eye. Keeping mosquitoes alive allows us to perform follow-up experiments to monitor the condition of mosquitoes in a time-dependent manner. As shown in Fig. 2, it was noted that longevity-related experiments are also possible. Third, any molecules, even insoluble substances that are smaller than the diameter of the food canal of mosquitoes (~ 30 μm) [25], can be added to the ATP solution harboring dyes. Given that we demonstrated the aversive effect of capsaicin on taste sensation in mosquitoes, novel taste attractants or repellants might be able to be identified using this approach. Lastly, different dyes can produce color-coded mosquitoes to identify each one individually. For example, two dyes could be applied to the two-choice test, which is a behavioral assay to investigate mosquito preference such as taste perception. It may result in observing two groups of mosquitoes with abdomens marked by different colors (see Fig. 1b).

Although ATP solution is a versatile phagostimulant buffer, it has an obvious limitation; it does not contain any nutrients such as amino acids, cholesterol, lipids, or metals, which are essential for triggering vitellogenesis followed by egg formation [5]. On the other hand, it appears that any dyes may fail to label the intact blood which is a dark shade of red. Recently, artificial blood diets were developed for rearing mosquitoes [23, 24, 26]. One of these is an ATP-supplemented plasma and effective even after extended storage in refrigerated, frozen, or lyophilized conditions. The color of plasma is generally translucent yellow. Using artificial blood diets with food dyes, we might be able to explore not only physiological but also reproductive mechanisms that require appropriate nutrition.

## Conclusions

In this study, we demonstrated that inclusion of food dyes improved the method for membrane feeding of ATP solution, in particular making it more feasible to estimate the volume sucked by a mosquito. Rough estimation using the naked eye may further expand the scope of experiments that need a blood-feeding process. ATP is a common phagostimulant among a number of hematophagous arthropods, and this method using food dye may be applicable for other blood-feeding species such as sandfly, tsetse fly, tick, louse, and flea, in addition to mosquitoes.

## Supplementary Information

**Additional file 1: Figure S1.** Equipment for membrane feeding used in this study. (a) Each glass feeder covered with stretched parafilm was placed on a paper cup containing approximately thirty female mosquitoes at 5–7 days after eclosion. After applying ATP solution into each feeder, 42 °C water from a hot water bath coupled with a water pump was circulated through rubber tubes connected to each feeder. (b) Magnified view of a paper cup with a glass feeder. Note that mosquitoes swarm around the feeder and suck the green colored solution.

**Additional file 2: Table S1.** List of solutions for membrane feeding used in this study.

**Additional file 3: Table S2.** Raw data of all experiments in this study.

## Data Availability

The datasets supporting the conclusion of this study are included within the article.
