# Supplementary figures and images for: A simple and affordable method for estimating the fluid volume a mosquito sucks using food dyes
Source: Trop Med Health. 2021 Feb 3;49:13. doi: 10.1186/s41182-021-00302-6 (PMC7856782; doi:10.1186/s41182-021-00302-6)

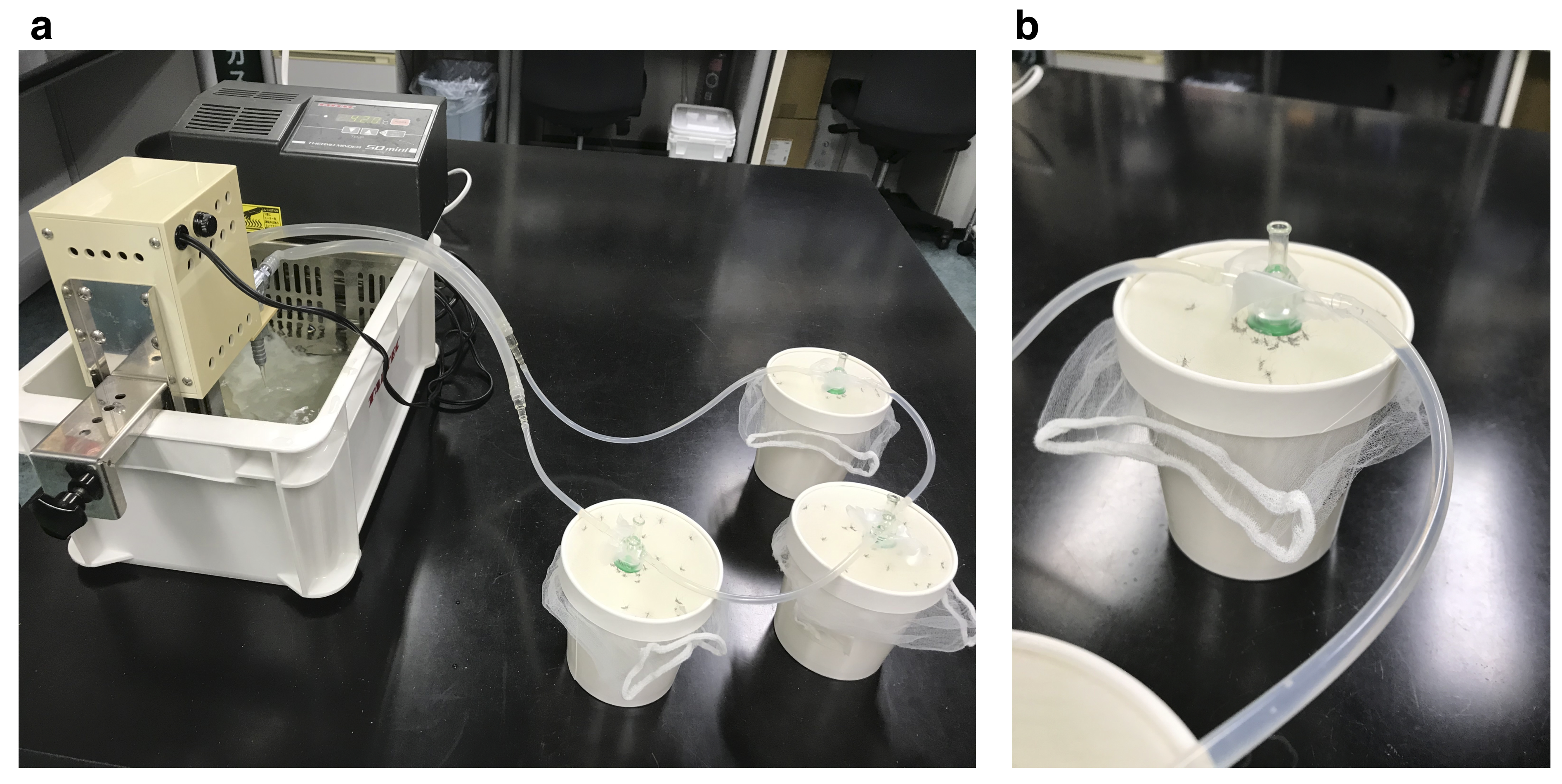

Supplement: Supplementary file 1 — Additional file 1: Figure S1. Equipment for membrane feeding used in this study. (a) Each glass feeder covered with stretched parafilm was placed on a paper cup containing approximately thirty female mosquitoes at 5–7 days after eclosion. After applying ATP solution into each feeder, 42 °C water from a hot water bath coupled with a water pump was circulated through rubber tubes connected to each feeder. (b) Magnified view of a paper cup with a glass feeder. Note that mosquitoes swarm around the feeder and suck the green colored solution. [file 41182_2021_302_MOESM1_ESM.tiff]
